# Evaluation of expressive writing for postpartum health: a randomised controlled trial

**DOI:** 10.1007/s10865-018-9970-3

**Published:** 2018-10-05

**Authors:** Susan Ayers, Rosalind Crawley, Susan Button, Alexandra Thornton, Andy P. Field, Chris Flood, Suzanne Lee, Andrew Eagle, Robert Bradley, Donna Moore, Gill Gyte, Helen Smith

**Affiliations:** 1 Centre for Maternal and Child Health Research, School of Health Sciences, City, University of London, Northampton Square, London, EC1V 0HB UK; 2 School of Psychology, University of Sunderland, Chester Road, Sunderland, SR2 7PT UK; 3 School of Psychology, University of Sussex, Brighton, BN1 9QH UK; 4 Central and North West London NHS Foundation Trust, London, W10 6DZ UK; 5 Brighton and Sussex University Hospital NHS Trust, Eastern Road, Brighton, BN2 5BE UK; 6 National Childbirth Trust, 30 Euston Square, London, NW1 2FB UK; 7 Division of Public Health and Primary Care, Brighton and Sussex Medical School, Brighton, BN1 9PH UK; 8 Lee Kong Chian School of Medicine, Nanyang Technological University, Singapore, 308232 Singapore

**Keywords:** Postpartum, Mental health, Physical health, Quality of life, Expressive writing, Self-help

## Abstract

Pregnancy, birth and adjusting to a new baby is a potentially stressful time that can negatively affect the health of women. There is some evidence that expressive writing can have positive effects on psychological and physical health, particularly during stressful periods. The current study aimed to evaluate whether expressive writing would improve women’s postpartum health. A randomized controlled trial was conducted with three conditions: expressive writing (*n* = 188), a control writing task (*n* = 213), or normal care (*n* = 163). Measures of psychological health, physical health and quality of life were measured at baseline (6–12 weeks postpartum), 1 and 6 months later. Ratings of stress were taken before and after the expressive writing task. Intent-to-treat analyses showed no significant differences between women in the expressive writing, control writing and normal care groups on measures of physical health, anxiety, depression, mood or quality of life at 1 and 6 months. Uptake and adherence to the writing tasks was low. However, women in the expressive writing group rated their stress as significantly reduced after completing the task. Cost analysis suggest women who did expressive writing had the lowest costs in terms of healthcare service use and lowest cost per unit of improvement in quality of life. Results suggest expressive writing is not effective as a universal intervention for all women 6–12 weeks postpartum. Future research should examine expressive writing as a targeted intervention for women in high-risk groups, such as those with mild or moderate depression, and further examine cost-effectiveness.

*Clinical trial registration number* ISRCTN58399513 www.isrctn.com

**Electronic supplementary material:**

The online version of this article (10.1007/s10865-018-9970-3) contains supplementary material, which is available to authorized users.

## Introduction

Approximately 136 million women every year give birth (World Health Organization, 2005). For the majority of women pregnancy and birth is positive, but some find the challenge of adjusting to the physical and emotional changes that accompany childbirth more difficult. Mental health problems such as postpartum depression, anxiety and post-traumatic stress can have a negative and enduring effect upon women and their families (Glasheen et al., 2010; World Health Organization, 2016). The World Health Organisation (WHO) lists mental illness as a significant indirect cause of maternal mortality (WHO, 2014). Maternal mental illness is associated with greater cognitive, behavioural and interpersonal problems in young children (Glasheen et al., 2010; Kingston & Tough, 2014). Recently, the cost of maternal mental illness to UK society was estimated at £8.1 billion per annual cohort of births, with 72% of this cost being due to the impact on children (Bauer et al., 2014).

Clinical guidelines emphasise the importance of early intervention but also highlight the lack of evidence-based interventions (National Institute for Health and Care Excellence, 2014). There is a need to develop evidence-based, low-risk interventions to improve physical and mental health, regardless of the type or severity of symptoms experienced. By targeting women soon after birth, postpartum interventions offer maximum scope for enhancing the wellbeing of women and children. In countries where healthcare is expensive and/or resources low it is also important that interventions are cost-effective.

Expressive writing could potentially improve women’s adjustment and health (Pennebaker & Chung, 2011) in a low-risk and cost-effective manner. Expressive writing interventions typically involve writing about one’s deepest thoughts and feelings about a particular stressful event for at least 15 min a day for 3 days (Pennebaker & Seagal, 1999; Smyth & Pennebaker, 2008). However, evidence for the effectiveness of expressive writing is mixed. Many studies have reported beneficial effects but the conclusions of recent meta-analyses are contradictory. Frattaroli (2006) reported small but significant beneficial effects of expressive writing on physical and psychological health. In contrast, Meads and Nouwen (2005) and Mogk et al. (2006) concluded current evidence has not clearly demonstrated its effectiveness but it may be beneficial for some health outcomes in certain contexts. This is supported by meta-analyses examining expressive writing within particular samples, or for specific outcomes. For example, Smyth (1998) reported a positive effect on physical and psychological functioning in healthy populations; Frisina et al. (2004) reported a small effect in clinical populations for physical health, but not psychological health; Harris (2006) concluded that it reduced healthcare utilisation in healthy but not clinical populations; and van Emmerik et al. (2013) concluded it is effective for reducing posttraumatic stress and comorbid symptoms of depression.

To date, few studies have focused on postpartum women, but the results are encouraging. Two studies found it was helpful for mothers of babies needing special care. Barry and Singer (2001) evaluated a non-standard form of expressive writing with women whose babies were in intensive care in the United States, and found that severe distress reduced from 37 to 16%. Similarly, Horsch et al. (2016) found that standard expressive writing reduced symptoms of posttraumatic stress and depression in mothers of very preterm infants. Other studies have examined the effect of writing about labour and birth: a series of studies by Di Blasio and colleagues found that women who wrote expressively about labour and birth the first week after birth had fewer symptoms of posttraumatic stress 2 or 3 months postpartum (Di Blasio & Ionio, 2002; Di Blasio et al., 2009; Di Blasio et al., 2015). Another study using a variation of expressive writing called a ‘making sense’ intervention (where mothers wrote about their labour and birth on one occasion in the first week postpartum) found women had fewer symptoms of depression and posttraumatic stress 3 months later than those who did not write (Di Blasio et al., 2015).

The results of these studies are promising but further investigation is needed (Peeler et al., 2013) as previous studies have focused on specific groups; used variations of the expressive writing paradigm; and many have insufficient power. This limits the extent to which results are informative about the effectiveness of expressive writing in the wider population of postpartum women and for a range of health outcomes. This paper reports a randomized controlled trial – the Health After Birth Trial (HABiT) – that evaluated the effect of expressive writing on postpartum mental health, quality of life and physical health; as well as the costs associated with health service use and change in health status.

## Method

### Design

HABiT was a parallel randomized controlled trial comparing expressive writing with a control writing task and normal care. The primary outcome was changes in mental health (mood, anxiety, depression). Secondary outcomes were changes in quality of life and physical health (physical symptoms, overall self-rated health). Women were randomized 6–12 weeks postpartum to one of three conditions: expressive writing, a control writing task, or normal care. Outcomes were measured pre-intervention (baseline), 1 and 6 months later. Costs were estimated using healthcare utilisation and quality of life data.

### Participants and procedure

Ethics permission was obtained from the National Health Service (NHS) Research Ethics Committee. Sample size calculations showed that to detect a small effect in primary outcome measures with a significance level of 0.05 and 80% power would require 122 women in each group, giving a total sample of 366 women. Participants were recruited through 14 NHS hospitals in England from November 2013 to December 2014. Women were eligible to participate if they were aged 18 years or older and had given birth to a live infant after 26 weeks gestation. Women who experienced stillbirth or neonatal death prior to hospital discharge were excluded, but women with current or previous psychological problems were not excluded.

All eligible women (*n* = 7986) in 14 NHS hospitals were invited to take part. Hospital based research staff put flyers in women’s discharge packs. Four to six weeks after birth they sent eligible women a letter inviting them to participate in the study, along with a participant information leaflet, consent form and reply paid envelope. Women could elect to participate by post or internet. Those who were willing to participate returned the consent form along with their contact details direct to the research team. Women who did not want to participate could reply giving their reasons if they wished to do so.

**Fig. 1:**
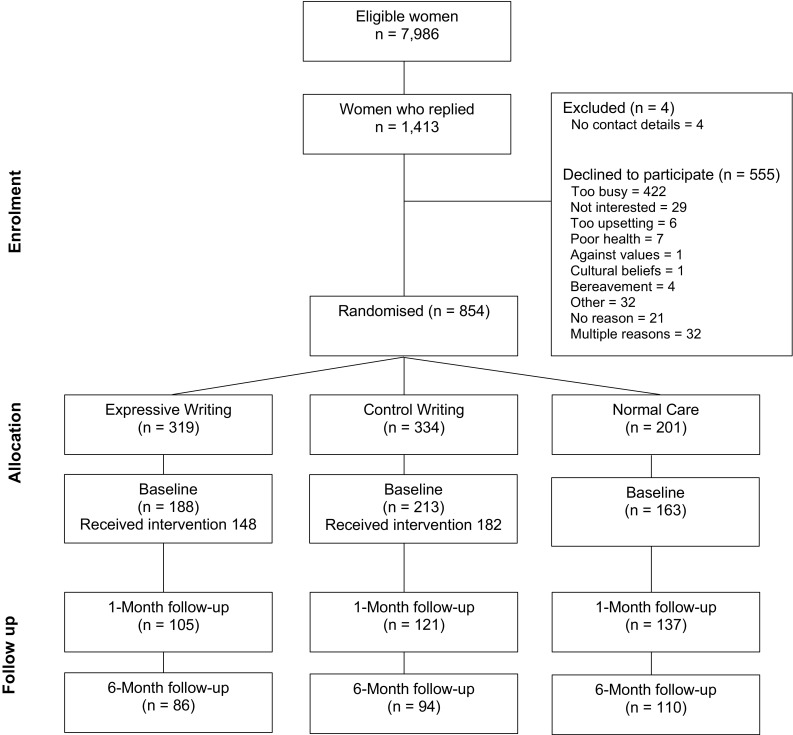
Sampling and attrition

Recruitment, allocation and sample attrition are shown in Fig. 1. Of the women approached, 1413 replied and 854 consented to take part (10.69% of eligible sample). Randomisation was initially on a 1:1:1 basis using a computerised random number generator. Initial attrition from the study was high, with 306 women failing to complete the first workbook. Some women (*n* = 16) went on to complete later measures, so baseline measures were imputed. The final sample for analyses was therefore 564 women. As dropout differed significantly between normal care and the expressive/control writing groups (Crawley et al., 2018), once there were enough participants in the normal care group all remaining participants were randomized on a 1:1 basis to the expressive writing or control writing conditions. There were no significant differences between women who dropped out from the expressive writing or normal care groups in age, parity or baseline measures of depression, anxiety, physical health and quality of life.

Following randomisation, women participating by post were sent workbook one with a reply-paid envelope. Reminders were sent by post, email or text message if workbooks were not returned within 10 days. Women participating via the internet were enrolled in the online system which generated an email or text message with their username and password. Those who did not log on and complete the workbook received further reminders after 7 days. Reminder times were slightly longer for postal participants to account for the delay incurred between postage and receipt of the workbooks. The majority of women who were randomized chose to complete the study online (63.2%). This did not differ between groups at the point of randomisation (χ^2^(2) = 1.65, *p* = .44) but did differ for women who completed the study with more women in the normal care group completing via post (χ^2^(2) = 7.55, *p* = .023).

All women completed baseline measures of mood, anxiety, depression, physical health, quality of life, and demographic measures (age, marital status, education, ethnicity, employment and previous psychological history). Women in the two writing conditions then completed the 3 day writing task, followed by additional measures of mood, anxiety and depression. Those in the expressive writing group also rated their stress before and after each writing session to check that writing had not increased stress. Follow-up measures of mood, anxiety, depression, physical health, and quality of life were collected for all groups at 1 and 6 months. Women in the control writing or normal care groups were offered the expressive writing intervention at the end of the study if they wished.

### Interventions

Normal postpartum care in the UK consists of daily checks by midwives and/or doctors whilst in hospital. After discharge, women are visited at home by a community midwife at least three times in the first 2 weeks (approximately 1, 5 and 10 days after discharge) or more if there are complications or issues that require monitoring. From 10 to 12 days postpartum, women are under the care of their general practitioner (GP) and have a routine check-up 6–8 weeks postpartum. Women are also assigned a community specialist nurse (health visitor) for maternal and child health from 0 to 5 years postpartum.

The expressive writing intervention was based upon Pennebaker’s expressive writing paradigm (Pennebaker & Seagal 1999; Smyth & Pennebaker, 2008). Women were instructed to write for 15 min each day about a stressful event related to their pregnancy, birth, baby, or something else going on in their life. Women were asked to write about their ‘deepest thoughts and feelings’ about this event. To avoid re-traumatising women the instructions stated that if writing about this event felt too distressing or overwhelming they should pick a less stressful event. Women were asked to complete all three writing exercises within a week, preferably on three consecutive days. Before and after writing women in the expressive writing condition were asked to rate ‘how upset or stressed are you by this event or difficulty now?’ on a 10-point scale from 1 (*not at all*) to 10 (*extremely*).

The control writing task was matched to the expressive writing task for time and basic structure. Women were asked to write about a familiar room, describing it objectively and not writing about feelings, beliefs or opinions. A measure of how clearly they could visualise the room from 1 (*not at all*) to 10 (*extremely*) was taken before and after the writing task. Women in both writing conditions were asked to indicate how distracted they had been whilst writing, and the date and time they started and stopped writing was recorded automatically online or by self-report in postal workbooks.

### Measures

The primary outcome was changes in psychological health. Secondary outcomes were changes in quality of life and physical health. Questionnaire measures had been previously used and validated with peripartum women. Psychological health was assessed in relation to mood, anxiety, depression, and mental health related quality of life. Participants also provided information about medication for a psychological condition and current or previous psychological problems. Physical health was evaluated using a symptom checklist, and physical health related quality of life. All measures are outlined below.

*Mood* was measured using the University of Wales Institute of Science and Technology Mood Adjective Checklist (Matthews et al., 1990). This consists of 24 mood adjectives. Participants indicate the extent to which each adjective describes their current mood on a 4-point scale (*definitely*, *slightly*, *slightly not*, *definitely not*). Some items are reverse scored and higher scores indicate more positive mood. The scale has good discriminant validity, is sensitive to stressors and has been used in female and postpartum samples. Internal reliability in our sample was high (α = .93).

*Mental Health* was measured using the Hospital Anxiety and Depression Scale (Zigmond & Snaith, 1983) which was designed to assess psychological distress in patient populations without being confounded by physical symptoms. It is therefore appropriate for postpartum women where physical symptoms such as fatigue are common. It comprises two 7-item subscales for anxiety and depression. Each item is scored from 0 to 3, with some items reverse-scored. Scores range from 0 to 21. Established cut-offs for the UK population are 8–10 for mild symptoms, 11–14 for moderate symptoms and 15 or more for severe symptoms. Internal reliability in our sample was acceptable (α = .83 anxiety, and α = .79 depression).

*Physical symptoms* were measured using the Physical Health Questionnaire-15 (Kroenke et al., 2002), a 15-item somatic symptom checklist designed to measure the severity of self-reported somatic symptoms and to screen for somatoform disorders. The extent to which each item has been bothersome over the previous two weeks is recorded on a 3-point scale (*not bothered*, *bothered a little*, *bothered a lot*). Higher scores indicate greater somatic symptom severity: cut off points are 5 (low), 10 (medium) and 15 (high) severity. Internal reliability in our sample was acceptable (α = .73) as were psychometric properties (Wilkie et al., 2017).

*Quality of Life* was measured using the Short Form Quality of Life questionnaire, version 2 (Ware et al., 2000), which is a widely used, standardised measure of quality of life with good internal reliability in obstetric samples. It comprises 12 questions about day-to-day functioning, scored on a 5-point or 3-point scale. Responses are imported into software purchased directly from the copyright holders which produces standardised z-scores for each participant. These were summed to provide physical health-related quality of life and mental health-related quality of life subscales. High scores indicate better quality of life. Internal reliability in our sample was acceptable (α = .86).

*Additional measures* Basic obstetric details were recorded from medical records. Use of healthcare services for non-routine visits to a general practitioner or hospital for women and their baby was measured by self-report at 1 and 6 months.

*Health costs* associated with each group were calculated based on healthcare service use (GP visits, adult and paediatric hospital admissions in days) as measured at the 1 month follow-up. Women were included if they completed measures of healthcare service use and quality of life (*n* = 342). Data were skewed by two very high cost outliers so these were removed.^1^ Unit cost data from published sources (Curtis & Burns, 2015; Department of Health, 2013) were attached to the resource use for each participant and multiplied to give total costs. Where necessary, estimated costs were adjusted to account for inflation. Once all resource use per participant had been calculated into a total health services cost this was placed alongside changes in participants’ physical and mental health quality of life scores from baseline to the 1-month follow-up, so the mean cost per unit of change in quality of life could be calculated to allow for comparison across all trial arms.

### Analysis

Analyses of demographic and outcome variables were conducted using R (R Core Team, 2016). Demographic variables were compared across treatment groups using Chi square tests for categorical variables (e.g., ethnic group), and a robust variant of a one-way ANOVA that corrects for heteroscedasticity by generalising the Welch method for continuous variables (e.g., age). The robust ANOVA was implemented using Wilcox’s (2012) *t1way* function from the WRS2 package (Mair et al., 2015; Wilcox, 2012). Bayes factors using default priors were computed using the BayesFactor package (Morey & Rouder, 2014) for each variable to quantify the relative evidence for the null against the alternative hypothesis. Values less than 1 indicate greater evidence for the null hypothesis (i.e., treatment groups did not differ).

The key outcomes were analysed using multilevel models in which observations (level 1) were nested within participants (level 2). For each outcome measure the model was built hierarchically. To begin with, an intercept only model (no predictors) was fit. Intercepts were then allowed to vary across participants (random intercept), which always improved the fit suggesting variability in mean levels of each outcome across participants. Next intercepts were allowed to vary across sites, but this addition never significantly improved the fit of the model and was never retained in the final model. The fixed effect of time (baseline, 1 month, 6 months) was added, and then allowed to vary across participants (random slopes). The random slope of time always improved the fit suggesting variable trajectories in all outcomes across participants. A first-order autoregressive covariance structure (AR(1)) was then imposed but in all but one model this model either did not converge, or did not improve the fit and so was not retained. Finally, the fixed effects of writing condition and its interaction with time was added. The final models can, therefore be represented as (*i* = individual at time *j*):

$$ Y_{ij} = \left[ {\gamma_{00} + \gamma_{10} {\text{Time}}_{ij} + \gamma_{01} {\text{Writing}}_{i} + \gamma_{11} \left( {{\text{Writing}}_{i} \times {\text{Time}}_{ij} } \right)} \right] + \left[ {\zeta_{0i} + \zeta_{1i} {\text{Time}}_{ij} + \varepsilon_{ij} } \right] $$

These models were fit using the lme() function from the nlme package (Pinheiro et al., 2015).

## Results

*Sample characteristics* are shown in Table 1. The sample was predominantly white European (94.7%), married or cohabiting (95.1%) and educated to degree level or higher (62.1%). The mean age of the participants was 32.77 years (*SD* = 5.38; range 18 to 46 years). The majority of participants were employed (*n* = 327; 83%) and a large proportion of these worked in a professional occupation as defined by the standard UK classification system (Office for National Statistics, 2010) (*n* = 132; 41.8%). There were no significant differences between intervention and control groups on any sociodemographic or baseline measures of outcomes.

**Table 1:** Sample characteristics

	Total Sample		Expressive Writing		Control Writing		Normal care		Test statistic	*p*
	*n*	%	*n*	%	*n*	%	*n*	%		
*Ethnicity*										
White European	373	94.7	102	94.4	118	92.2	153	96.8	Fisher’s exact test = 8.27	.338
African	3	0.8	1	0.9	1	0.8	1	0.6		
Asian	5	1.3	3	2.8	2	1.6	–	–		
Mixed	5	1.3	–	–	3	2.3	2	1.3		
Other	8	2.0	2	1.9	4	3.1	2	1.3		
	394		108		128		158			
*Relationship status*										
Married	250	64.8	66	62.3	76	60.8	108	69.7		
Cohabiting	117	30.3	36	34.0	42	33.6	39	25.2		
Separated/divorced	4	1.0	2	1.9	–	–	2	1.3	Fisher’s exact test = 9.82	.198
Single	13	3.4	2	1.9	7	5.6	4	2.6		
Other	2	0.5	–	–	–	–	2	1.3		
	386		106		125		155			
*Gestation*										
26 < 32 weeks	9	1.7	3	1.7	2	1	4	2.5	Fisher’s exact test = 3.15	.800
32 < 37 weeks	24	4.4	5	2.8	11	5.4	8	5.1		
37 < 40 weeks	251	46.3	84	46.9	95	46.3	72	45.6		
> 40 weeks	258	47.6	87	48.6	97	47.3	74	46.8		
	542		179		205		158			
*Parity*										
Nulliparous	254	46.9	87	48.3	97	47.5	70	44.3	χ^2^(2) = .61	.737
Multiparous	288	53.1	93	51.7	107	52.5	88	55.7		
	542		180		204		158			
*Type of birth*										
Normal vaginal	323	60.8	112	63.6	115	57.2	96	62.3	*χ*^2^(6) = 3.47	.748
Assisted vaginal	70	13.2	25	14.2	27	13.4	18	11.7		
Emergency caesarean	78	14.7	22	12.5	35	17.4	21	13.6		
Elective caesarean	60	11.3	17	9.7	24	11.7	19	12.3		
	531		176		201		154			
*Complications*										
None	221	42.9	72	42.4	76	39.6	73	47.4	*χ*^2^(8) = 7.62	.472
Maternal complications	145	28.1	45	26.5	59	30.7	41	26.6		
Infant complications	83	16.1	34	20	28	14.6	21	13.6		
Maternal and infant complications	67	13	19	11.2	29	15.1	19	12.3		
	516		170		192		154			
*Education level*										
None	4	1	2	1.9	1	0.8	1	0.6	Fisher’s exact test =	.899
GCSE/O	49	12.7	11	10.3	16	13.1	22	14.1		
A-Level	93	24.2	29	27.1	28	23	36	23.1		
Degree +	239	62.1	65	60.7	77	63.1	97	62.2		
	385		107		122		156			
*Employment*										
Yes	327	83	90	83.3	103	80.5	134	84.8	*χ*^2^(2) = .960	.620
No	67	17	18	16.7	25	19.5	24	15.2		
	394		108		128		158			
*Diary writing*										
Regularly	14	3.6	3	2.8	4	3.1	7	4.5	Fisher’s exact test = 2.56	.870
Sometimes	53	13.5	14	13	21	16.4	18	11.5		
Rarely	63	16	19	17.6	21	16.4	23	14.6		
Not at all	263	66.9	72	66.7	82	64.1	109	69.4		
	393		108		128		157			

*Manipulation checks and adherence* Analyses of the tasks and adherence are reported in detail elsewhere (Crawley et al., 2018). These showed the intervention was effective in terms of content of writing. Writing groups did not differ in the number of words written or time they took to write, but did differ on content: expressive writing participants used significantly more emotional and cognitive processing words, but fewer perceptual words. However, adherence to the full writing protocol (to write for 15 min on three days) was low with only 29.3% of women in the expressive writing group and 23.5% of women in the control writing group complying with these instructions. Adherence to the writing task was not predicted by type of writing task (control vs. expressive writing), anxiety or depression at baseline, education level, complications during birth, parity, or mental health-related quality of life at baseline. There were no significant differences between writing groups in the potential confounding factor of how distracted women were during the writing tasks (Crawley et al., 2018).

*Effectiveness of expressive writing on health outcomes* Table 2 shows the model parameters for the multilevel models (see earlier description) for each health outcome. The models were parameterized so that the main effect of group was dummy coded comparing expressive writing (the baseline) to normal care and control writing. The main effects of group in these models show the effect of expressive writing on health outcomes. This shows that expressive writing had no significant effect on mood, anxiety, depression or quality of life. There was a trend for an effect of group on overall levels of physical symptoms (*p* = .051), but change over time was not moderated significantly by the writing condition. Similar results were found when analyses were restricted to women who adhered to the writing tasks (i.e. wrote for at least 15 min a day on 3 days). Women who adhered were more likely to have depression and physical symptoms at baseline.

**Table 2:** Effectiveness of expressive writing

	*β*	SE	*t* Value	*df*	*P*	*β*	SE	*t* Value	*df*	*P*
	**Anxiety**					**Depression**				
Intercept	6.43	.30	21.64	636	**< .001**	4.98	.26	19.07	636	**< .001**
1 month follow up	− .59	.26	− 2.31	636	**.022**	− .74	.25	− 2.95	636	**.003**
6 months follow up	− .36	.34	− 1.05	636	.295	− .48	.38	− 1.26	636	.209
Expressive writing versus Control writing	.28	.41	.68	550	.496	.11	.36	.31	550	.756
Expressive writing versus Normal care	− .29	.43	− .66	550	.508	− .56	.38	− 1.46	550	.145
1 month follow up: EW versus Control writing	.21	.35	.59	636	.556	.38	.34	1.12	636	.264
6 months follow up: EW versus Control writing	.39	.47	.82	636	.414	.26	.53	.51	636	.614
1 month follow up: EW versus Normal care	.45	.34	1.32	636	.187	.38	.33	1.14	636	.254
6 months follow up: EW versus Normal care	.36	.46	.80	636	.423	.18	.51	.35	636	.724
Model fit: Time	χ^2^ − 3101.08	AIC 6212.16	BIC 6237.59	5	**.046**	χ^2^ − 3050.94	AIC 6111.87	BIC 6137.30	5	**.009**
Model fit: Group	χ^2^ − 3087.32	AIC 6198.65	BIC 6259.68	12	.378	χ^2^ − 3020.67	AIC 6065.34	BIC 6126.37	12	.064
Model fit: Group x Time interaction	χ^2^ − 3086.24	AIC 6204.47	BIC 6285.85	16	.704	χ^2^ − 2019.81	AIC 6071.62	BIC 6153.00	16	.789
	**Quality of Life (Mental Health)**					**Quality of Life (Physical Health)**				
Intercept	41.41	.44	93.21	624	**< .001**	55.6	.68	82.12	622	**< .001**
1 month follow up	− 1.33	.54	− 2.44	624	**.015**	3.68	.69	5.33	622	**< .001**
6 months follow up	− 2.42	.62	− 3.91	624	**< .001**	4.9	.89	5.51	622	**< .001**
Expressive writing versus Control writing	− .13	.61	− .21	538	.834	− .28	.93	− .30	538	.767
Expressive writing versus Normal care	.04	.64	.06	538	.951	.70	.98	.71	538	.479
1 month follow up: EW versus Control writing	.05	.74	.06	624	.949	− .32	.95	− .34	622	.733
6 months follow up: EW versus Control writing	1.04	.85	1.22	624	.224	− 1.3	1.22	− 1.06	622	.290
1 month follow up: EW versus Normal care	.23	.74	.31	624	.753	− .30	.94	− .32	622	.750
6 months follow up: EW versus Normal care	.13	.85	.16	624	.874	− 1.29	1.21	− 1.06	622	.289
Model fit: Time	χ^2^ − 3609.10	AIC 7228.20	BIC 7253.53	5	**< .001**	χ^2^ − 4004.84	AIC 8019.68	BIC 8045.00	5	**< .001**
Model fit: Group	χ^2^ − 3598.70	AIC 7221.39	BIC 7282.18	12	.922	χ^2^ − 3973.42	AIC 7972.84	BIC 8038.67	13	.413
Model fit: Group x Time interaction	χ^2^ − 3597.38	AIC 7226.77	BIC 7307.82	16	.622	χ^2^ − 3972.57	AIC 7979.14	BIC 8065.23	17	.790
	**Mood**					**Physical Symptoms**				
Intercept	5.56	1.22	44.82	604	**< .001**	7.13	.31	23.29	616	**< .001**
1 month follow up	− 1.11	1.05	− 1.06	604	.290	− .92	.29	− 3.19	616	**.002**
6 months follow up	− .32	1.39	− .23	604	.817	− 1.24	.42	− 2.93	616	**.004**
Expressive writing versus Control writing	.52	1.67	− .32	525	.754	.22	.42	.52	532	.601
Expressive writing versus Normal care	3.21	1.76	1.82	525	.069	− .52	.44	− 1.16	532	.247
1 month follow up: EW versus Control writing	1.85	1.44	1.29	604	.199	.66	.40	1.66	616	.096
6 months follow up: EW versus Control writing	1.59	1.92	.83	604	.406	.59	.58	1.01	616	.311
1 month follow up: EW versus Normal care	− .26	1.4	− .18	604	.856	− .05	.39	− .13	616	.898
6 months follow up: EW versus Normal care	− .31	1.85	− .17	604	.869	.86	.57	1.52	616	.130
Model fit: Time	χ^2^ − 4544.55	AIC 9099.10	BIC 9124.28	5	.689	χ^2^ − 3089.62	AIC 6189.23	BIC 6214.50	5	**.001**
Model fit: Group	χ^2^ − 4522.99	AIC 9069.98	BIC 9130.43	12	.179	χ^2^ − 3070.06	AIC 616412	BIC 6224.76	12	**.051**
Model fit: Group x Time interaction	χ^2^ − 4521.53	AIC 9075.05	BIC 9155.64	16	.570	χ^2^ − 3066.19	AIC 6164.38	BIC 6245.23	16	.101

Bold values indicate outcome measure

*EW* expressive writing

Change over time was observed for most outcomes. Overall levels and change varied across participants but this change was not significantly moderated by writing condition. Anxiety and depression reduced over time, with a significant decrease observed at 1 month but not at 6 months. The quality of life mental health subscale scores changed over time, with significant decreases observed at 1 month and 6 months, suggesting women’s quality of life related to mental health worsened over time. Physical symptoms significantly improved over time, as did quality of life related to physical health.

Ratings of how stressed women in the expressive writing group were by the event they wrote about were analysed with a multilevel model in which ratings (level 1) were nested within women (level 2). Fixed effects of time (before vs. after writing), day (day 1, 2, or 3) and their interaction were included. Intercepts for stress varied significantly across women (*LR* = 470.02, *p* < .001) and there were significant main effects of time (*LR* = 7.44, *p* = .006), and day (*LR* = 105.18, *p* < .001), but not their interaction (*LR* = 2.43, *p* = .30). Model parameters showed that stress levels were significantly lower on day 2 than day 1 (*b* = −0.69, *SE* = 0.12, *t*(590) = −5.50, *p* < .001), and on day 3 compared to day 1 (*b* = −1.39, *SE* = 0.13, *t*(590) = −10.70, *p* < .001).

*Potential moderators* Unplanned exploratory analyses were conducted to see how the effect size for expressive writing compared to normal care changed as a function of participants’ baseline anxiety and depression scores. This was achieved by fitting a model to compare expressive writing to normal care in subsets of participants defined by threshold levels of depression or anxiety at baseline, and then systematically increasing that threshold to examine effect sizes for each point on the subscale. For example, scores on the depression subscale at baseline ranged from 0 to 14 (from a possible range of 0 to 21). No participants had severe depression at baseline (i.e. a score of 15 or more). We began by setting the threshold at 0 and fitted the model including all 544 participants (*ns* = 180 expressive writing, 204 control writing, 160 normal care). The threshold then moved to 1 (i.e. excluded cases with baseline depression scores of 0) yielding a model based on 519 cases (*ns* = 169 expressive writing, 198 control writing and 152 normal care), then to 2 (i.e. excluding cases with baseline depression below this value) to yield a model based on 455 cases (*n*s = 142 expressive writing, 177 control writing and 136 normal care), and so on until the threshold was 12 and the model included only those 26 participants who scored 12 or more at baseline (*n*s = 7 expressive writing, 12 control writing and 7 normal care).

Results suggest that the effect of expressive writing was greatest in women who had mild to moderate symptoms of depression at baseline (i.e. a score of 9 or more; see Figure, Supplemental Digital Content 1). However, this finding needs to be treated very cautiously because (1) it was not planned a priori; (2) as the threshold level of depression for inclusion increases the sample size decreases, therefore, the apparent influence of baseline depression is confounded by the lack of precision with which we can estimate the effect of expressive writing; and (3) the subsample of women with a depression score of 9 or more at baseline was small (*ns* = 36 expressive writing, 30 control writing and 18 normal care), and estimates are more variable in small samples. As such, this finding requires replication. Levels of anxiety at baseline did not affect the effect of expressive writing.

*Cost analysis* The mean cost associated with health service resource use in different groups was: £517 for expressive writing, £721 for control writing, and £657 for normal care. This is a saving of £140 compared to normal care (or 19% of costs of normal care). When mean cost data was considered alongside mean change in physical health quality of life for each group, the associated cost per unit of improvement was £138 for the expressive writing group, £192 for the control writing group and £201 for the normal care group: a saving of £63 compared to normal care (or 31% of costs of normal care). Mean costs per unit of change in mental health showed a similar pattern, with the cost per unit of change being lowest for the expressive writing group (£346) and highest for the normal care group (£570). However, changes in scores on the mental health quality of life subscale from baseline to the 1-month follow up were small so this should be interpreted with caution.

## Discussion

The HABiT trial aimed to examine the efficacy of expressive writing for improving postpartum health. Results show expressive writing was not effective as a universal intervention for women 6–12 weeks after birth. However, in the expressive writing group stress associated with the event they wrote about significantly decreased after writing. Cost analysis suggest women who did expressive writing had the lowest costs in terms of healthcare service use and costs per unit of improvement in quality of life. Exploratory moderator analyses suggested expressive writing may be more effective in women with mild to moderate depression but this requires replication.

These results are consistent with some studies in non-obstetric samples. Meta-analyses of the effects of expressive writing report mixed findings (Frattaroli, 2006; Meads & Nouwen, 2005; Mogk et al., 2006; Smyth, 1998; Frisina et al., 2004; van Emmerik et al., 2013). Some conclude that although participants who write often feel it is beneficial the evidence does not clearly demonstrate its effectiveness (Meads & Nouwen, 2005; Mogk et al., 2006). The finding that expressive writing was associated with lowest costs for healthcare service use is consistent with the meta-analysis by Harris (2006) which found that expressive writing reduced healthcare service use in healthy populations but not clinical populations.

Mogk et al. (2006) acknowledge that expressive writing may be beneficial for some health outcomes in certain contexts. In HABiT, women were more likely to adhere to the writing tasks if they had greater physical symptoms and depression. Similar results have been found in other studies (Broderick et al., 2004). Possible explanations include that people with greater symptoms at baseline are more motivated to adhere to expressive writing; more likely to benefit; and/or that expressive writing is more likely to be effective when fully adhered to.

The results of HABiT are inconsistent with previous studies with postpartum women, where all the published research to date has found positive benefits of expressive writing (Barry & Singer, 2001; Horsch et al., 2016; Di Blasio & Ionio, 2002; Di Blasio et al., 2009; Di Blasio et al, 2015; Di Blasio et al., 2015). A few of these studies sampled high-risk women who are likely to be distressed i.e. women with babies born preterm or in NICU (Barry & Singer, 2001; Horsch et al., 2016), whereas HABiT used systematic sampling to try to get a representative, normative cohort. It is therefore possible that expressive writing is more likely to be effective when it is targeted at particular groups of high-risk women. Exploratory threshold analysis of HABiT data provided some support for this, with a suggestion that the effect of expressive writing may be greater in women with mild to moderate depression scores at baseline. However, as there were very few women in this sample with mild to moderate depression this analysis requires replication.

Timing of the intervention is also likely to be important, as the demands of caring for a new baby may make it difficult for women to find time to write regularly without distractions, especially in the early postpartum period. In previous studies women either wrote in the first week, often whilst they were still in hospital (Di Blasio & Ionio, 2002; Di Blasio et al., 2009; Di Blasio et al., 2015; Di Blasio et al., 2015), or after 3 months postpartum (Barry & Singer, 2001; Horsch et al., 2016). In HABiT women were recruited at 4–6 weeks postpartum and asked to complete the writing task 6–12 weeks postpartum, which may have been a factor in the low uptake and adherence. The acceptability and feasibility of expressive writing to postpartum women at different times and in different contexts may help explain our inconsistent findings. Crawley et al. examine the feasibility and acceptability of expressive writing in HABiT and conclude that the feasibility of using expressive writing as a universal intervention for all women 6–12 weeks after birth is low because of the poor uptake and high levels of dropout. However, for women who use expressive writing it is an acceptable intervention (Crawley et al., 2018).

Outcome measures should also be considered. Previous studies with postpartum women focused on psychological outcomes of posttraumatic stress, distress and depression, and found positive effects of expressive writing. HABiT extended these findings by examining quality of life, physical symptoms and costs associated with healthcare service use and improved quality of life. However, HABiT did not include a measure of posttraumatic stress because such symptoms were not expected to be common in a normative sample. A meta-analysis of expressive writing for posttraumatic stress in multiple populations concluded it is effective for reducing posttraumatic stress and comorbid symptoms of depression (van Emmerik et al., 2013). This may be an important outcome to include in future research with high-risk postpartum women.

Although expressive writing did not improve health outcomes when used as a universal intervention for all women, it also did not do harm. Women who did the expressive writing task found it acceptable, reported reduced stress about the event they wrote about, and were generally positive about expressive writing (Crawley et al., 2018). This is consistent with previous literature. For example, a meta-analysis of emotional disclosure which found no positive effects of emotional disclosure on a range of health outcomes also observed that there were no negative effects (Meads et al., 2003). Thus, if expressive writing is offered as one of a range of self-help interventions then women who self-select to do it (for whatever reason) may be more likely to adhere and gain benefit from it even if it does not improve health outcomes. Further research is needed to examine this.

Expressive writing was also associated with the lowest healthcare costs. The results suggested that, compared to normal care, expressive writing was associated with a 19% saving in healthcare service use and a 31% saving in costs per unit of improvement in physical health quality of life compared to normal care. Similar findings were also observed for costs for mental health. This is consistent with meta-analyses which find that expressive writing reduces healthcare service use in healthy populations but not clinical populations (Harris, 2006; Meads et al., 2003). Harris (2006) suggests that expressive writing may address concerns in people who use healthcare services frequently by helping them explore and satisfy their concerns, thus reducing use of healthcare services. In HABiT many women wrote about health concerns with the baby or themselves so it is possible that this acted to reduce healthcare service costs. Of course, it is impossible to determine whether this is positive or negative in terms of health outcomes, only that it reduces healthcare service costs. Further research is needed to establish the cost-effectiveness of expressive writing with postpartum women, the mechanisms underlying this effect, and what impact it has on health outcomes.

### Strengths and limitations

Strengths of this study include being the first study to evaluate expressive writing as a universal intervention for all postpartum women. It is also the largest randomized controlled trial examining expressive writing in this population to date. Outcomes measures were carefully chosen to be valid in this population. Limitations are the low uptake and adherence rates, which shows the feasibility of using expressive writing so early in the postpartum period is poor. The low uptake also means the sample is not representative of the population, with a high proportion of white European women educated to degree level or above. Results are therefore not generalizable to all postpartum women and future research is needed with women from ethnic minority groups or lower levels of education.

### Implications for research and practice

This study has a number of implications for clinical practice. A major consideration is the use of expressive writing (and perhaps self-help interventions generally) as a universal or targeted intervention. Universal interventions are applied to all women in a prophylactic manner with the aim of aiding adjustment and positive health. This approach in HABiT resulted in low uptake and high dropout. There are many possible explanations for this, such as the intervention being offered too early postpartum, as discussed above. In addition, it could be speculated that women who did not have problems adjusting postpartum, or who had severe problems adjusting, may have been less motivated to take part.

In contrast, targeted interventions are offered to women with specific characteristics as a form of prevention or treatment, such as in previous studies of expressive writing for women with preterm babies (Horsch et al., 2016), or the possibility suggested here of offering expressive writing to women with mild to moderate depression after birth. The results of HABiT clearly show the universal application of expressive writing is not warranted or feasible in the early postpartum period. However, there are many possible reasons for this and further research is needed to examine it as a targeted intervention for high-risk women.

There are also implications for research. This discussion has outlined some of the ways in which sampling, timing of interventions, type of writing task and outcome measures may all influence whether expressive writing is effective for postpartum women. Future research should consider sampling high-risk groups, offering the intervention when women have time, such as whilst in hospital or after 3 months postpartum, including outcome measures of posttraumatic stress, and conducting further cost analyses of healthcare service use.

## Conclusion

In conclusion, this study shows that expressive writing is not effective as a universal intervention for women 6–12 weeks after birth. These results are consistent with some studies of expressive writing in other populations (Meads & Nouwen, 2005; Mogk et al., 2006), but not consistent with research with postpartum women (Barry & Singer, 2001; Horsch et al., 2016; Di Blasio & Ionio, 2002; Di Blasio et al., 2009; Di Blasio et al., 2015; Di Blasio et al., 2015). This is probably due to methodological differences such as sampling and timing of the intervention. However, expressive writing was associated with reduced self-rated stress and healthcare use and costs. This is consistent with meta-analyses showing expressive writing is associated with reduced healthcare use in healthy samples (Harris, 2006; Meads et al., 2003).

Poor uptake and adherence to the writing tasks suggests expressive writing is not feasible for many women at this time (Crawley et al., 2018). Women who adhered to the expressive writing task had more physical symptoms and depression so may have been more motivated to complete it, although symptoms of depression were very mild in this sample and within the normal range. There is some suggestion that expressive writing may be more effective in women with mild to moderate depression. Future research should therefore examine expressive writing as a targeted intervention for women in high risk groups, such as those with mild to moderate depression, and look at the mechanisms underlying reduced healthcare costs and any impact this has on health outcomes.

## Electronic supplementary material

Below is the link to the electronic supplementary material.

Effect sizes of expressive writing for women according to baseline scores of depression and anxiety (PDF 13 kb)

## Footnotes

1 These outliers were a participant whose baby was in the neonatal intensive care unit for 104 days (expressive writing group) and a woman admitted to hospital after birth (normal care group).
