# Supplementary figures and images for: Evaluation of expressive writing for postpartum health: a randomised controlled trial
Source: J Behav Med. 2018 Oct 5;41(5):614–26. doi: 10.1007/s10865-018-9970-3 (PMC6209049; doi:10.1007/s10865-018-9970-3)

b for Contrast

EW vs.CW

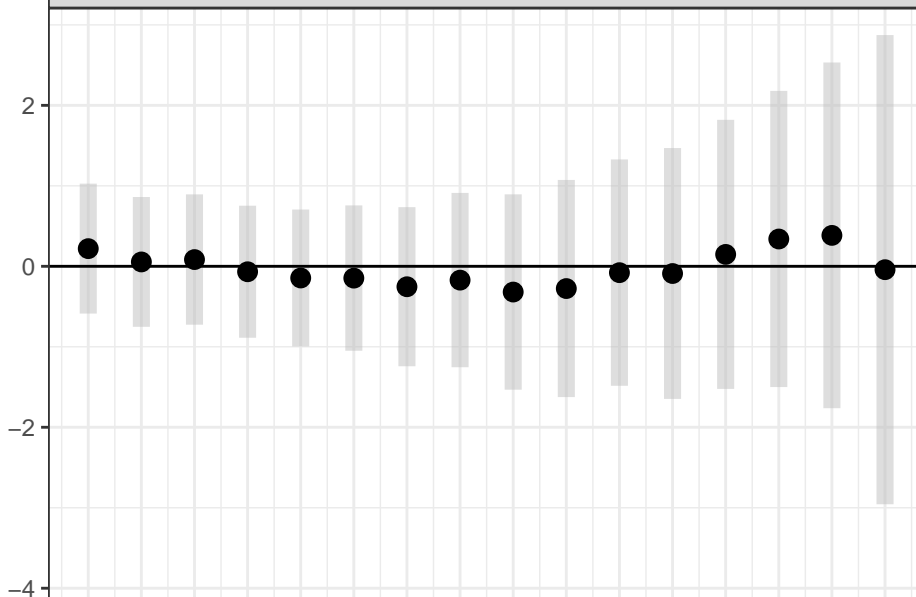

EW vs. NC

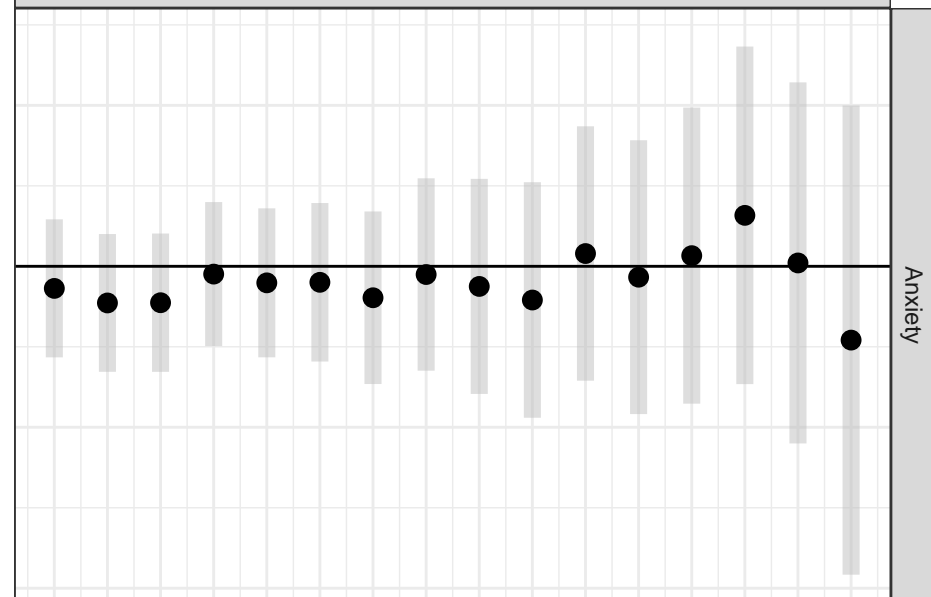

Anxiety

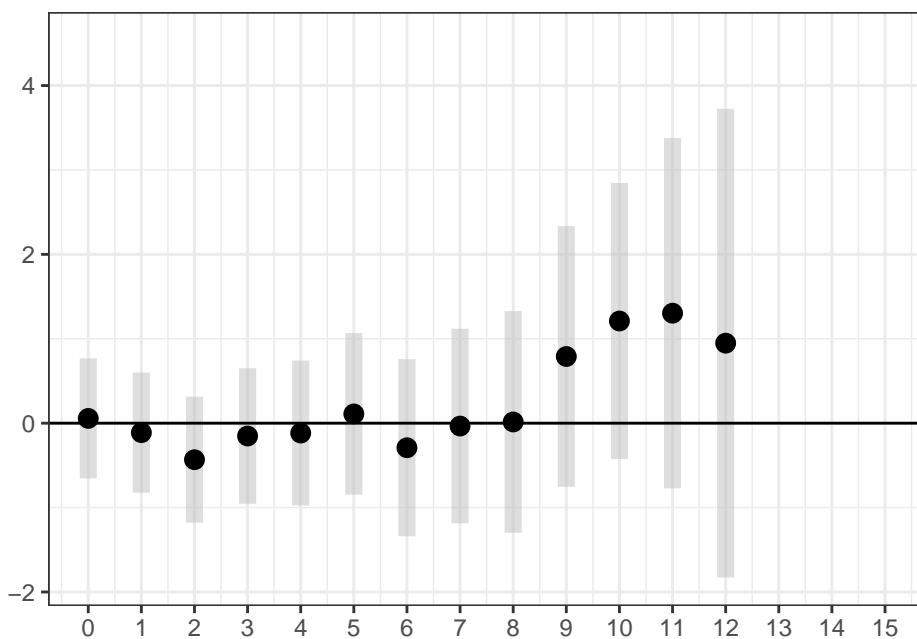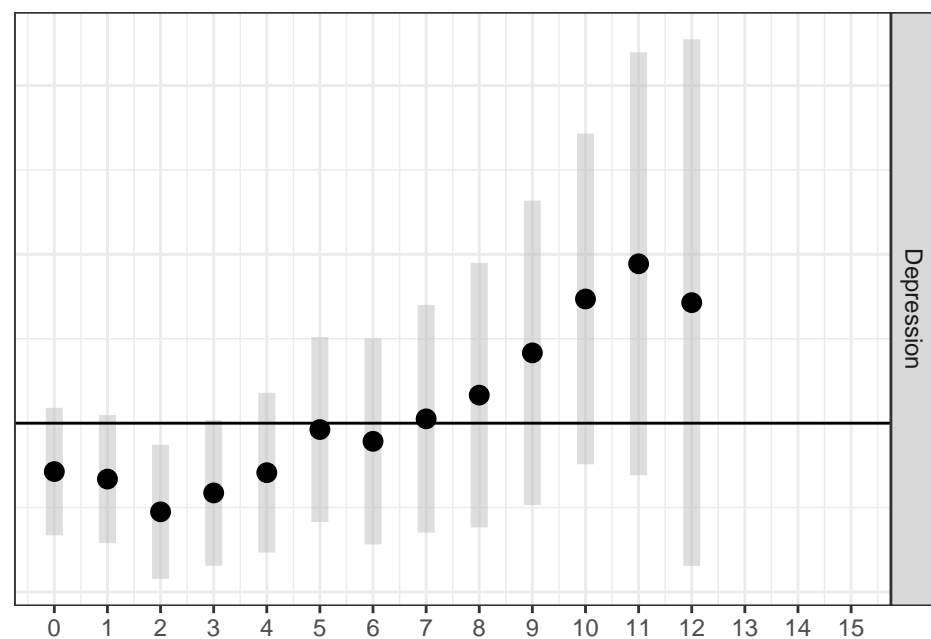

Depression

Threshold

Supplement: Supplementary file 1 — Effect sizes of expressive writing for women according to baseline scores of depression and anxiety (PDF 13 kb) [file 10865_2018_9970_MOESM1_ESM.pdf]
